# Cognitive functioning and hyperhomocysteinemia in schizophrenia

**DOI:** 10.1016/j.scog.2025.100411

**Published:** 2025-12-17

**Authors:** Ushasti Sinha, Shambhavi Shiva, Anirudh Rajan, Abhiram Narasimhan Purohith, Praveen Arahanthabailu, Rajesh Krishna Bhandary, P.S.V.N. Sharma

**Affiliations:** a Department of Psychiatry, Kasturba Medical College, Manipal, Manipal Academy of Higher Education, Manipal, Karnataka, India -576104

**Keywords:** Cognitive functions, Homocysteine/blood, Schizophrenia/blood, Vitamin B-complex/therapeutic use

## 1 Introduction

Schizophrenia is among the top ten causes of global disability, and beyond positive symptoms, improvement in negative and cognitive symptoms determines the extent of independent living and real-world functioning (Bowie and Harvey, 2006). Hyperhomocysteinemia has been implicated as a risk factor for schizophrenia in the extant literature (Muntjewerff et al., 2006). While the direct role of Homocysteine (Hcy) in the complex pathogenesis of schizophrenia is hypothetical, it is evident that elevated Hcy levels have been associated with higher severity of negative and cognitive symptoms (Trześniowska-Drukała et al., 2019; Levine et al., 2006; Roffman et al., 2013). The evidence on folate-vitamin B12 supplementation and homocysteine reduction strategies on cognitive symptoms in schizophrenia is heterogeneous (Trześniowska-Drukała et al., 2019; Levine et al., 2006; Roffman et al., 2013). We aim to report significant improvement in cognitive functions and negative symptoms in a patient with treatment-resistant schizophrenia after correcting homocysteinemia. Written informed consent has been obtained from the patient to publish this report.

## 2 Case description

Mr. VR is a 43-year-old single gentleman diagnosed with treatment-resistant schizophrenia with an illness duration of 18 years. He was staying at our psychiatric rehabilitation centre for the past two years for vocational rehabilitation. He was currently under a supported employment program. His medications included a stable dose of Clozapine 500 mg/day, Phenytoin 300 mg/day and Flupenthixol long-acting injectable 40 mg/month. The psychotic symptoms were in remission. Co-morbidities were type-2 diabetes mellitus and hypertriglyceridemia, for which he was on Metformin 500 mg/day and Fenofibrate-Atorvastatin, respectively. Glycaemic control was adequate. Family history is significant for ischemic heart disease, peripheral vascular disease, and dyslipidaemia. As part of the periodic evaluation of cardiac risk, serum homocysteine was assessed along with other biochemical tests and an electrocardiogram. Lipid control was inadequate, and serum homocysteine was elevated (41.5 μmol/L; normal range 5–15 μmol/L), while other tests were normal, including normal B12 and folate levels.

Clinical Global Impression Scale – Schizophrenia (CGI-SCH) (Haro et al., 2003) was used to assess global clinical impression at baseline and after Hcy-reduction. A detailed neurocognitive assessment was done before and after. The tests included digit span for working memory, trail making test A and B (TMT-A and TMT—B) for processing speed and executive functioning, categorical fluency, and phonemic fluency. A digitised version of the Stroop test from the psychology experiment building laboratory (PEBL) test battery was used to assess cognitive flexibility and response inhibition (Mueller and Piper, 2014). Similarly, a digitised version of the Tower of London (TOL) test from the PEBL battery was used to assess planning and problem-solving abilities (Mueller and Piper, 2014). Vitamin B12, B6 and folate were supplemented both parenterally and orally. Hcy level after six months was 15.9 μmol/L.

There was an improvement in the global impression of negative symptoms and cognitive performance, especially executive functioning & cognitive flexibility (TMT-A&B performance). In the Stroop test, a Wilcoxon signed-rank test showed a statistically significant change in congruent response time (W = 289, *p* < 0.001) and incongruent response time (W = 285, p < 0.001). Accuracy of responses improved from 98.6 % to 100 %. This indicates improvement in cognitive flexibility. In the TOL test, there was an improvement in the average execution time from 46.2 s to 43.4 s after correcting Hcy. However, this was not statistically significant. Details are summarized in Table 1. Although a formal assessment has not been done, there was an improvement in performance at his work as reflected by the feedback from his employer.

**Table 1:** Summary of performance on various cognitive tests and CGI-SCH before and after correcting homocysteine levels.

Cognitive tests		Baseline	After reduction of Hcy
Digit span	Digit Forward	6	6
	Digit Backward	4	5
Categorical fluency	Animals	12	11
	Vegetables	9	10
	Food items	12	11
Phonemic fluency	Pa	7	8
	Ka	8	8
	Ma	9	9
TMT - A	Time	80 s	75 s
	Errors	No error	No error
TMT-B	Time	247 s	226 s
	No of errors	1 error	No error
Stroop test	Congruent RT	4506 ms	2205 ms
	Incongruent RT	6808 ms	2776 ms
	Interference score	2302	571
TOL test	Average execution time	46.2 s	43.4 s
CGI-SCH	Positive symptoms	2 - Minimally ill	1 – Very much improved
	Negative symptoms Depressive symptoms	3 - Mildly ill 3 - Mildly ill	2 – Much improved 1 – very much improved
	Cognitive symptoms	3 - Mildly ill	2 – Much improved
	Global severity	3 - Mildly ill	2 – Much improved

## 3 Discussion

In this report, we have highlighted the improvement in negative symptoms and cognitive functioning, including executive functioning, cognitive flexibility, and working memory, with the correction of Hcy levels in a patient with TRS. Hcy plays an integral role in cognitive and behavioural processes through its direct interaction with glutamate receptors and indirect interaction with the monoaminergic neurotransmitter system. Increased Hcy levels have been linked with oxidative stress, neuronal apoptosis, excitotoxicity and vascular damage (Diaz-Arrastia, 2000). Hyperhomocysteinemia has been widely reported in patients with schizophrenia and is usually underrecognized. Serum Hcy level has been shown to correlate with the severity of positive and negative symptoms in schizophrenia, whereas effects on Hcy reduction on cognitive performance are mixed (Trześniowska-Drukała et al., 2019; Roffman et al., 2013; Petronijević et al., 2008). While the evidence on the benefits of Hcy-lowering treatments on cognition is heterogeneous in healthy subjects (Setién-Suero et al., 2016), there is limited research on the benefits in schizophrenia.

Although the improvement of response time on TMT is modest and could be due to practice effect, the improvement in response time and errors in the Stroop test is significant. This is in contrast with earlier reports, where there was no improvement on these tests (Trześniowska-Drukała et al., 2019; Levine et al., 2006). This could be attributed to the beneficial effects of prolonged supplementation with B_12_, B_6_ and folate in our patient compared to shorter duration of supplementation in earlier studies (Trześniowska-Drukała et al., 2019; Levine et al., 2006). Clinical impression of improvement in negative symptoms was also comparable to the extant reports (Trześniowska-Drukała et al., 2019; Roffman et al., 2013; Setién-Suero et al., 2016). This could be attributed to both improvement in homocysteine levels as well as folate levels, as earlier research has demonstrated the inverse correlation between serum folate level and severity of negative symptoms (Goff et al., 2004).

Although the confounding effects of non-pharmacological interventions at our centre could have played a role, no new interventions were initiated during this period, as he was in a stable job. Hyperhomocysteinemia could be due to several factors, including Vitamin B_12_ or folate deficiency, polymorphism of the methylenetetrahydrofolate gene, obesity, excessive smoking or dietary deficiency (Levine et al., 2006). Some of these confounding factors were absent in our patient. It is currently unclear whether routine Vitamin B_12_ and folate supplementation would be beneficial in the absence of hyperhomocysteinemia in schizophrenia (Levine et al., 2006; Roffman et al., 2013).

## 4 Conclusion

There is current evidence to indicate that increased homocysteine can mediate variables that could impair prognosis through increased negative and cognitive symptoms in schizophrenia. Further investigation into the effectiveness of routine screening and long-term supplementation is warranted.

## CRediT authorship contribution statement

**Ushasti Sinha:** Formal analysis, Data curation. **Shambhavi Shiva:** Data curation. **Anirudh Rajan:** Supervision, Formal analysis, Data curation, Conceptualization. **Abhiram Narasimhan Purohith:** Writing – review & editing, Writing – original draft, Supervision, Methodology, Investigation, Formal analysis, Data curation, Conceptualization. **A. Praveen:** Writing – review & editing, Supervision. **Rajesh Krishna Bhandary:** Writing – review & editing. **P.S.V.N. Sharma:** Writing – review & editing.

## Declaration of Generative AI and AI-assisted technologies in the writing process

None used.

## Declaration of competing interest

The authors declare that they have no known competing financial interests or personal relationships that could have appeared to influence the work reported in this paper.
